# Clinical presentation and outcome of gastric impactions with or without concurrent intestinal lesions in horses

**DOI:** 10.1111/jvim.16735

**Published:** 2023-07-04

**Authors:** Sophie E. Talbot, Rose Tallon, Bettina Dunkel

**Affiliations:** ^1^ Department of Clinical Science and Services The Royal Veterinary College Hatfield UK; ^2^ Donnington Grove Veterinary Group Newbury UK

**Keywords:** colic, dietary management, equine, gastric rupture, recurrence, stomach

## Abstract

**Background:**

Gastric impactions (GI) have been identified as primary lesions (lone GI; LGI) or associated with other intestinal lesions (concurrent GI; CGI). Anecdotally, CGI resolve more rapidly with a better prognosis than LGI.

**Objectives:**

To determine clinical, laboratory, and ultrasonographic findings, and short‐ and long‐term survival in horses with GI. We hypothesized that LGI carries a worse prognosis than CGI.

**Animals.:**

Seventy‐one horses from 2 referral hospitals (2007‐2022).

**Methods:**

Retrospective cohort study. Gastric impactions were defined as feed extending to the margo plicatus after ≥24 hours of fasting. Clinical, diagnostic and outcome findings were compared between LGI and CGI. Long‐term survival was determined by a questionnaire.

**Results:**

Twenty‐seven horses had LGI, 44 had CGI. Large intestinal lesions (32/44) were more common than small intestinal lesions (12/44). Concurrent gastric impactions resolved more slowly than LGI (LGI median 2 days, range 0‐8; CGI median 4 days, range 1‐10; *P* = .003). Short‐ (LGI 63%, 17/27; CGI 59%, 26/44; *P* = .75) and long‐term survival (LGI 3.5 ± 1.9 years; CGI 2.3 ± 2.3 years; *P* = .42) were not significantly different. However, Lone gastric impactions were more likely to experience gastric rupture (LGI 29.6%, 8/27; CGI 11.4%, 5/44; *P* = .05). Lone gastric impactions were 8.7 times more likely to require dietary changes (LGI 72.7%, 8/11; CGI 25%, 4/16; 95% confidence interval [CI], 1.53‐49.22; *P* = .01). Gastric impactions recurred in 21.7% (LGI, 6/20; CGI, 4/26; *P* = .23) of affected horses.

**Conclusions and Clinical Importance:**

Lone gastric impactions and CGI present similarly with a comparable prognosis, but LGI are more likely to rupture. Long‐term dietary changes are often necessary for horses with LGI.

**Abbreviations**
CI: confidence interval
CGI: concurrent gastric impactions
GI: gastric impactions
IACUC: Institutional Animal Care and Use Committee
LGI: lone gastric impactions
Se: sensitivity
Sp: specificity
SSRERB: Social Sciences Research Ethical Review Board
TNCC: total nucleated cell count
TP: total protein

## 1 INTRODUCTION

Gastric impactions (GI) are a well‐recognized but poorly understood cause of colic in horses. They have been defined as persistent, progressive accumulations of dehydrated ingesta that remain within the stomach after prolonged fasting, often defined as ≥24 hours.^1^ Classification systems used to describe GI include acute vs chronic or primary vs secondary, if an inciting cause can be identified.^1^ However, GI often progresses insidiously initially with few clinical signs, and chronicity can be difficult to establish. Feed accumulation can occur after ingestion of expansive, poorly digestible, or excessive feed and has been associated with hepatic disease, particularly pyrrolizidine alkaloid toxicosis.^2^, ^3^, ^4^ However, GI also can occur without any apparent inciting cause^5^ and has been observed in the presence of other gastrointestinal lesions, such as large colon displacements or volvuli.^6^ Anecdotally, GI occurring concurrently with other intestinal lesions (concurrent gastric impactions [CGI]) appear to resolve more rapidly and carry a better prognosis compared with cases where GI are the only abnormality identified (lone gastric impactions [LGI]). Diagnosis of either condition can be challenging because clinical signs are often vague, ranging from weight loss to poor performance or colic.^5^

The primary aim of our retrospective cohort study was to describe and compare clinical and diagnostic findings, response to treatment and short‐ and long‐term outcomes in horses with LGI and CGI. We tested the hypothesis that treatment of horses with LGI took longer and carried a worse short‐ and long‐term prognosis than did treatment of those with CGI.

## 2 MATERIALS AND METHODS

Clinical records from 2 equine referral centers were searched for horses diagnosed with GI from 2007 to 2022. Gastric impactions were defined as presence of feed material within the stomach, extending to the level of the margo plicatus, after at least 24 hours of fasting. Cases were excluded if the fasting period was <24 hours or if horses had known dental or hepatic disease. Horses <1 year old and donkeys also were excluded. A definitive diagnosis was achieved by gastroscopy, palpation of the stomach during exploratory laparotomy or necropsy. Confirmed cases were divided into CGI or LGI, depending on the presence or absence of concurrent intestinal lesions suspected of causing signs of colic.

Clinical and laboratory information extracted from the case records included patient signalment, history, presenting clinical signs and laboratory findings. In cases of CGI, the nature of the concurrent lesion was noted. This classification was based on the interpretation of diagnostic or surgical findings by the attending clinician. Reports of ultrasonographic examinations, particularly stomach size but also descriptive abnormalities, were collated and compared if available. A gastric silhouette visible over ≥6 intercostal spaces during ultrasonographic examination was considered enlarged.^7^, ^8^ Gastroscopy reports were assessed and a subjective measure of impaction size during gastroscopy was recorded: moderate GI were defined as feed material extending to the level of the margo plicatus, whereas large GI were defined as feed extending above the margo plicatus. If feed material extended to the level of the cardia or into the esophagus, GI were defined as very large. If horses underwent exploratory laparotomy, surgical findings including a subjective assessment of stomach size as described in the surgeon's report (normal, moderate, large, or very large) were included. Ultrasonographic size estimates were compared with surgical or gastroscopic size estimates. Sensitivity (Se) and specificity (Sp) of ultrasonographic gastric silhouette enlargement (≥6 intercostal spaces) were calculated to determine whether ultrasonographic size could be used to predict the size of GI as determined at surgery or gastroscopy. If present, gastric ulcers were recorded, both descriptively and scored on a scale of 4, based on recommended guidelines.^9^, ^10^ Treatment methods and response to treatment were noted, including volume of IV and enteral fluids administered, use of carbonated soft drinks or prokinetics, time to GI resolution as determined by gastroscopy, time to discharge, and any recurrence during hospitalization. Short‐term survival was defined as survival to discharge.

Owners were contacted using an online or telephone questionnaire to evaluate long‐term outcome, recurrence of a GI or colic signs, and any management changes implemented. Ethical approval was obtained from the Social Sciences Research Ethical Review Board (SSRERB) of the Royal Veterinary College.

### 2.1 Statistical analysis

Data were analyzed using commercially available software (IBM SPSS Statistics 28.0.0.0). Normality of the data was assessed using Shapiro‐Wilk tests. Continuous data were represented as mean ± SD if normally distributed, or median and range (minimum‐maximum) if not normally distributed. Categorical data were represented as numbers and percentages. Chi‐squared tests and Fisher's exact tests were used to compare categorical measurements. Comparisons between parametric continuous data were made using a Student's *t*‐test; nonparametric data comparisons were made using Mann‐Whitney *U* tests. Odds ratios were represented with 95% confidence intervals (CI). Significance was set at *P* ≤ .05.

## 3 RESULTS

### 3.1 Case signalment, history, and presenting signs

Seventy‐one horses met the inclusion criteria; 38% (27/71) of cases were categorized as LGI and 62% (44/71) as CGI. The study population included 64.8% (46/71) geldings, 33.8% (24/71) mares and 1.4% (1/71) stallions. The mean age was 14 ± 5.6 years (n = 65); 6 horses were of unknown age. Twenty different breeds were represented including Warmblood and cross breeds (n = 15), Thoroughbreds and cross breeds (n = 9), Irish sport horses and cross breeds (n = 5), Welsh ponies (n = 5), Irish draught and cross breeds (n = 5), Cobs and cross breeds (n = 5), Arabians and cross breeds (n = 3), Shetland ponies (n = 3), ponies (n = 2), Connemaras and cross breeds (n = 2), New Forest ponies (n = 2), and 1 each of Polo ponies, Trotter horses, Andalusians, Selle Francais, Friesian, and Shires. Nine horses were of unknown breed. No significant differences in sex or age distribution were found between the 2 groups (Table 1).

**TABLE 1:** Signalment, clinical and laboratory parameters of lone, concurrent, and total cases upon admission.

	Lone Gastric Impactions	Concurrent Gastric Impactions	All cases	P value
Age	14 ± 6.9 (n = 25)	14 ± 4.7 (n = 40)	14 ± 5.6 (n = 65)	*P* = .66
Sex	19 geldings, 1 stallion, 7 mares (n = 27)	27 geldings, 17 mares (n = 44)	46 geldings, 24 mares, 1 stallion (n = 71)	*P* = .27
Heart rate (beats per minute)	47 ± 3.1 (n = 25)	56 ± 26.0 (n = 41)	53 ± 22.8 (n = 66)	*P* = .13
Respiratory rate (breaths per minute)	17 (8‐68) (n = 24)	17 (12‐60) (n = 34)	17 (8‐68) (n = 58)	*P* = .84
Temperature (°C)	37.9 ± .8 (n = 23)	37.5 ± .6 (n = 35)	37.7 ± .7 (n = 58)	*P* = .03
Packed cell volume (%)	38 ± 6.1 (n = 24)	40 ± 8.7 (n = 41)	39 ± 7.9 (n = 65)	*P* = .34
Total protein (TP) concentration (g/L)	69 ± 6.9 (n = 24)	66 ± 8.2 (n = 40)	67 ± 7.8 (n = 64)	*P* = .24
Systemic lactate concentration (mmol/L)	1.7 (.7‐13.2) (n = 11)	1.9 (.5‐15.1) (n = 36)	1.7 (.5‐15.1) (n = 47)	*P* = .45
Serum amyloid A concentration	290 (2‐1330) (n = 7)	279 (76‐2080) (n = 3)	285, (2‐2080) (n = 10)	*P* = .67
WBC (×10^9^/L)	6.5 (4.0‐15.4) (n = 12)	7.4 (3.3‐24.7) (n = 14)	6.7 (3.3‐24.7) (n = 26)	*P* = .46
Peritoneal fluid total nucleated cell count (TNCC) (×10^9^/L)	1.2, (.2‐17.0) (n = 8)	.8, (.2‐34.8) (n = 17)	1, (.2‐34.8) (n = 25)	*P* = .55
Peritoneal fluid TP (g/L)	7 ± 19.9 (n = 6)	14 ± 14.5 (n = 17)	18 ± 15.7 (n = 23)	*P* = .61
Peritoneal fluid lactate concentration (mmol/L)	6.2 ± 7.8 (n = 5)	7.0 ± 8.6 (n = 14)	3.8 ± 8.2 (n = 19)	*P* = .86

Abbreviation: TNCC, total nucleated cell count; TP, total protein concentration; WBC, white blood cell count.

The most common reason for the presentation was acute colic (71.8%, 51/71), followed by chronic or recurrent colic (8.5%, 6/71). Other reasons included chronic esophageal obstruction, poor performance, pyrexia, anorexia, and weight loss. The median duration of clinical signs was 12 hours (1‐1344 hours). The horse that presented after 56 days (1344 hours) was excluded from the analysis involving the duration of clinical signs, because the clinical signs were intermittent in nature and the specific onset of signs before presentation could not be determined in this horse. Removing this horse, the median duration of clinical signs was 10 hours (1‐672 hours). The duration of clinical signs before presentation was significantly longer in the LGI group than in the CGI group (LGI, 24 hours; range, 1‐672 hours; CGI, 6 hours; range, 1‐96 hours; *P* = .01). Seven percent (5/71) had experienced gastric rupture on arrival, which was confirmed by exploratory laparotomy (n = 3) or necropsy (n = 2). Of these, 60.0% (3/5) were classified as LGI and 40.0% (2/5) as CGI (*P* = .3).

### 3.2 Clinical variables

Clinical and laboratory variables at presentation are presented in Table 1.

### 3.3 Diagnostic tests

Ultrasonographic measurements of the gastric silhouette were available in 26/71 (36.6%) cases. Of these gastric silhouettes, 24% (15/26) were considered enlarged, extending to ≥6 intercostal spaces. Ultrasonographically enlarged gastric silhouettes were not significantly associated with a large or very large size estimate of gastric impaction during surgery (*P* = .29) or during gastroscopy (*P* = 1.00). Using presence of large or very large GI during surgery as the gold standard, ultrasonographic findings had poor sensitivity and specificity of 67% and 20%, respectively, when used to diagnose GI. When comparing ultrasonographic enlargement with the presence of large or very large GI on gastroscopy, sensitivity was 55% and specificity was 50%. Rectal examination was performed in 54/71 (76.1%) cases (LGI, 70.3%, 19/27; CGI, 79.5%, 35/44). Rectal examination was performed in all horses with CGI that did not undergo immediate exploratory laparotomy or euthanasia. Of the LGI group, rectal examination was not performed in 4 horses; 3 of these did not present for colic signs. Two of these horses were referred for treatment of GI and 1 was referred for recurrent esophageal obstruction but gastroscopy identified an extensive GI. The fourth horse was a Shetland Pony and rectal examination was not performed because of its small size.

The initial diagnosis of a GI was established by gastroscopy in 32/71 (55.2%) cases, during surgical exploration of the abdomen in 34/71 (47.8%) cases, and at necropsy in 5/71 (7%) cases. In cases diagnosed during surgery, gastroscopy was later performed in 26/34 cases (76.5%): in 19/26 (73.0%) cases GI persisted, whereas in 7/26 (26.9%) it had resolved. The other 8/34 horses were subsequently euthanized. Overall, gastroscopy was performed in 58/71 (81.7%) cases (LGI, 37.9%, 22/58; CGI, 62.1%, 36/58). The median fasting period was 24 hours (range, 24‐168 hours). No significant differences were found in gastroscopic findings, including gastric impaction size or presence of other gastric disease between groups (Table 2).

**TABLE 2:** Comparison of impaction size at gastroscopy, equine squamous gastric disease grade, and equine glandular gastric disease grading between LGI, CGI, and total cases.

	LGI	CGI	Total	*P*‐value
Impaction size estimated during gastroscopy	5/22 moderate 17/22 large	7/36 normal, 7/36 moderate, 20/36 large, 2/36 very large	7/58 normal 12/58 moderate 37/38 large 2/36 very large	*P* = .09
Grade of squamous lesions following impaction clearance (out of 4)	15/20 grade 0	22/30 grade 0	37/50 grade 0	*P* = .66
	1/20 grade 1	0/30 grade 1	1/20 grade 1	
	1/20 grade 2	3/30 grade 2	4/50 grade 2	
	1/20 grade 3	1/30 grade 3	2/50 grade 3	
	2/20 grade 4	4/30 grade 4	6/50 grade 4	
Grade of glandular lesions following impaction clearance (out of 4)	14/20 grade 0	26/30 grade 0	40/50 grade 0	*P* = .12
	1/20 grade 1	0/30 grade 1	1/20 grade 1	
	2/20 grade 2	0/30 grade 2	2/50 grade 2	
	1/20 grade 3	1/30 grade 3	2/50 grade 3	
	2/20 grade 4	3/30 grade 4	5/50 grade 4	

Six horses developed GI postoperatively, 3/6 (50%) were diagnosed by gastroscopy, 2/6 (33%) by necropsy, and 1/6 (16.7%) at both gastroscopy and necropsy.

Necropsy was performed in 19/71 (26.8%) cases (LGI, 22.2%, 6/27; CGI, 30.2%, 13/43) and GI was confirmed in all but 1 horse (18/19). This horse was euthanized after GI resolution because of persistent colic signs, secondary to extensive adhesions throughout the abdomen. Gastric rupture was identified in 8/19 (42.1%) cases, 1/19 (5.2%) had a GI and duodenal rupture. Thirteen of 71 (18.3%) horses experienced gastric rupture. The LGI horses were significantly more likely to experience gastric rupture than CGI horses (LGI, 29.6%, 8/27; CGI, 11.4%, 5/44; *P* = .05).

### 3.4 Diagnosis of concurrent intestinal lesions

Concurrent intestinal lesions are summarized in Table 3. The most common concurrent intestinal lesions were large colon volvuli, comprising 24.4% (11/44) of cases, followed by small intestinal strangulating lesions comprising 11.1% (5/44), and right dorsal displacements 8.9% (4/44) of cases. One horse had a small strangulating lipoma and large colon impaction concurrently with a GI. Including large colon impactions, displacements, and volvuli, large intestinal lesions were more common than small intestinal lesions (large intestinal lesions, 72.3%, 32/44; small intestinal lesions, 27.3%, 12/44).

**TABLE 3:** Concurrent intestinal lesions identified in the study population.

Lesion type	Number of lesions recorded in the population	Percentage of concurrent lesions (%)
Large colon torsion	11	25
Small intestinal obstruction (strangulating)	5	11.4
Right dorsal displacement	4	9.1
Large colon impaction (unspecified)	3	6.8
Pelvic flexure impaction	3	6.8
Ileal obstruction/strangulation	3	6.8
Large colon fluid distension or colitis	3	6.8
Large colon displacement (unspecified)	3	6.8
Inflammatory bowel disease	2	4.5
Small intestinal obstruction (nonstrangulating)	1	2.3
Right dorsal colon displacement and impaction	1	2.3
Left dorsal displacement and impaction	1	2.3
Large colon displacement and impaction (unspecified)	1	2.3
Cecal impaction	1	2.3
Small colon impaction	1	2.3
Duodenal rupture	1	2.3
Total	44	100

### 3.5 Exploratory laparotomy

Fifty‐five percent of all cases (39/71) consisting of 11.1% LGI (3/27) and 81.8% CGI (36/44) underwent exploratory laparotomy; 34/39 (87.1%) surgeries identified a GI (the remaining 5 cases developed postoperatively). Twenty‐five percent (9/34) were described as moderate GI, 28.6% (10/34) as large, and 42.8% (15/34) as very large.

A significant association was found between the size of the GI estimated at surgery and euthanasia (moderate impactions comprising 11.1% [1/9] of those euthanized; large and very large impactions comprising 60.0% [15/25] of those euthanized; *P* = .03).

### 3.6 Treatment

Eighty‐five percent (60/71) of horses underwent treatment for GI. Of the horses that were treated, 16.7% (10/60) received enteral fluid therapy only (all LGI), 18.3% (11/60) received IV fluid therapy only (LGI 18.2%, 2/11; CGI 81.8%, 9/11) and 37/60 received both (LGI 24.3%, 9/27; CGI 75.7%, 28/37). Twenty‐seven percent (16/60) received carbonated, caffeine‐ and sugar‐free soft drink in combination with enteral fluid therapy (18.8% [3/16]), IV fluid therapy (6.3% [1/16]), or both (68.8% [11/16]). Prokinetics were not used in any horse during initial treatment. Because of the retrospective nature of the study, information regarding volumes of enteral fluids or carbonated soft drinks was not available. Of the horses receiving IV fluids, the median volume of fluids administered was 60 L (10‐170 L).

During hospitalization, 19.7% of horses (14/71) experienced recurrent colic signs, more frequently in CGI cases than in LGI cases (LGI, 3.8%, 1/26; CGI, 29.5%, 13/44; *P* = .01). Median time to resolution of GI from the first day in hospital was 3 days (range, 0‐10 days). The CGI took significantly longer to resolve compared with LGI (LGI median, 2 days; range, 0‐8 days; CGI median, 4 days; range, 1‐10 days; *P* = .003). Subjective evaluation of GI size at surgery (26 large GI, 57.7%, 15/26 died; *P* = .02) but not gastroscopy (39 large GI, 33.3%, 13/39 died; *P* = .52) was significantly associated with short‐term survival to discharge. One horse with CGI developed a second GI during hospitalization after attempted refeeding.

### 3.7 Short‐ and long‐term survival

The percentage overall short‐term survival to discharge was 60.6% (43/71), which was not significantly different between LGI (63.0%, 17/27) and CGI (59.1%, 26/44; *P* = .75). Mean hospitalization time was 6.8 ± 2.9 days and did not differ between LGI (5 ± 3.2 days) and CGI (6 ± 3.8 days; *P* = .27).

Of the 31 horses that did not survive, 28/31 (90.3%) were euthanized and 3/31 (9.7%) died; 14/31 (45.2%) of these horses were euthanized within the first 24 hours of presentation. Fourteen of 31 (45%) including 6/14 (42.9%) of the horses euthanized within 24 hours succumbed to fecal peritonitis secondary to gastric rupture, diagnosed at surgery or necropsy. Gastric rupture was more common in LGI than CGI (LGI 29.6%, 8/27; CGI 11.4%, 5/44; *P* = .05). Long‐term follow‐up was available for 30/71 (42.3%) cases, consisting of 11/27 (40.7%) of LGI and 19/44 (43.2%) of CGI cases. Time since discharge ranged from 9 months to 10 years (3.7 ± 2.8 years). Of the 43 horses discharged, 9/43 (20.9%) were re‐admitted to the hospital for a recurrence of GI (LGI 66.7%, 6/9; CGI 33.3%, 3/9; *P* = .06). At long‐term follow‐up, 5/28 (17.9%) were reported to have experienced a recurrence of GI (LGI 60%, 3/5; CGI 40%, 2/5; *P* = .31). Combining these groups, the overall rate of recurrence was 21.7% (10/46 cases recorded; LGI 30%, 6/20; CGI 15.4%, 4/26; *P* = .23). Recurrence was not significantly different between LGI and CGI.

Overall, 56.7% (17/30) of horses were still alive (LGI 45.5%, 5/11; CGI 63.2%, 12/19), whereas 12/30 (40%) had died (LGI 54.5%, 6/11; CGI 31.6%, 6/19). Two horses were sold shortly after discharge (both CGI cases), 1 was known to still be alive and the other was lost to follow‐up; both were excluded from analysis. Causes of death included colic (30%; 4/12), laminitis (40%; 3/12), chronic weight loss unresponsive to treatment (16.7%; 2/12), failure of gastric ulcer treatment (8.3%; 1/12), acute neurological disease (8.3%; 1/12), and fracture (8.3%; 1/12). The average number of years between discharge and death was 2.9 ± 2.1 years and the average age of death was 20.8 ± 7.1 years. Neither number of years between discharge and death (LGI 3.5 ± 1.9 years; CGI 2.3 ± 2.3 years; *P* = .42) nor average age at death (LGI 22.75 ± 4.8 years; CGI 19.50 ± 8.7 years; *P* = .52) were significantly different between LGI and CGI.

After discharge, 50.0% (14/28) of horses returned to a normal diet and exercise regimen. The remaining 50% (14/28) required ongoing management changes (Table 4). The LGI horses were 8.7 times more likely to require altered dietary management compared with CGI horses (LGI 72.7%, 8/11; CGI 25%, 4/16; 95% CI: 1.53‐49.22; *P* = .01). Altered diets varied from soaked short‐fiber diets, grass only diets to partial‐ or complete‐pelleted diets. The LGI cases were significantly more likely to require a partial‐ or complete‐pelleted diet compared with CGI cases (LGI 54.5%, 6/11; CGI 12.5%, 2/17; *P* = .01). No significant difference in return to normal exercise was found (LGI 40%, 2/5; CGI 64.7%, 11/17; *P* = .18). Five horses (LGI 27.2%, 3/11; CGI 11.8%, 2/17) still were being treated with metoclopramide; 40% (2/5) at the time of follow up, 40% (2/5) at the time of euthanasia. Metoclopramide was discontinued in 1 horse 4 months after discharge. This horse experienced intermittent recurrence of clinical signs despite maintenance on a partial‐pelleted diet and was eventually euthanized, because of a suspected gastric rupture.

**TABLE 4:** Long‐term dietary, medication, and exercise outcomes for lone gastric impactions (LGI), concurrent gastric impactions (CGI), and total cases.

	LGI	CGI	Total	*P*‐value
Return to normal diet	3/11 (27.3%)	13/17 (76.4%)	16/28 (57.1%)	*P* = .01
Partial or complete pelleted diet	6/11 (27.3%)	2/17 (11.8%)	8/28 (28.6%)	*P* = .014
Use of metoclopramide	3/11 (27.3%)	2/17 (11.8%)	5/28 (17.9%)	*P* = .295
Return to normal exercise	5/11 (45.5%)	12/17 (70.6%)	17/28 (60.7%)	*P* = .184
Return to normal exercise and diet	3/11 (27.3%)	11/17 (64.7%)	14/28 (50.0%)	*P* = .05

## 4 DISCUSSION

Although horses with LGI tended to show clinical signs for a longer period before presentation, horses with LGI and CGI both presented with a very similar clinical picture, supporting the hypothesis that LGI and CGI have a similar etiology and essentially represent the same disease process. Judging GI as very large during surgery correlated significantly with nonsurvival, but size assessments using other diagnostic modalities (e.g., ultrasonography, gastroscopy) did not. Short‐ and long‐term survival were similar for horses with LGI and CGI, but horses with LGI were significantly more likely to experience gastric rupture and require long‐term dietary modification, which might be associated with the longer duration of clinical signs before diagnosis and subsequent treatment.

Gastric impactions in horses are not uncommon, but the literature on the condition remains sparse. Definition of GI as dehydrated feed material extending to the level of the margo plicatus is widely accepted,^1^, ^11^ but the fasting time necessary before residual feed material can be called an impaction is debated. Some investigators have suggested shorter fasting periods of 12 to 16 hours^5^, ^12^ whereas others chose a 16‐hour fasting period.^5^ To minimize the chances of including horses with delayed gastric emptying rather than true impaction, a fasting time of at least 24 hours was used in our study. To avoid confounding extragastrointestinal conditions that have been associated with GI, cases with hepatic disease were excluded.^2^ Donkeys and foals also were excluded because gastric emptying in donkeys is anecdotally reported to be slower, and foals could suffer from gastric outflow obstruction secondary to duodenal ulceration, which is assumed to be a different disease process.^13^

The presence of GI with another intestinal lesion only recently has been described, raising questions of whether GI occur first, potentially triggering an acute intestinal incident or whether GI are possibly secondary to concurrent intestinal lesions. The similar clinical presentation, response to treatment and short‐ and long‐term outcomes identified in our study suggest that the GI are the primary lesions with very similar etiology in LGI and CGI. However, other forms of colic also present with similar clinical signs despite different causes and it cannot be excluded that the 2 forms of GI are distinct diseases, despite their apparent similarities. Colon torsions previously have been associated with GI^6^ and were the most common concurrent intestinal lesion in our population, comprising 25% of the CGI group. Large intestinal lesions were numerically more common (31/44 lesions) than small intestinal lesions, and it is possible that the space‐occupying nature of GI could predispose to the development of these lesions, particularly large colon displacements or torsions. Brood mares and taller horses carry a significantly higher risk for large colon volvulus.^14^ The increased risk is thought to be associated with a change in intraabdominal potential space in postpartum mares. The increased volume taken up by gastric enlargement has been suggested as contributing factor in the pathogenesis of large colon volvulus^15^ and our findings seem to support this hypothesis.

Although some clinical features differed between the LGI and CGI, the overall presentation and response to treatment were remarkably similar. Clinical and laboratory variables were largely comparable. Although rectal temperature was significantly higher in the LGI group, temperature remained within the normal range, and the difference was not clinically relevant. As might be expected, LGI cases had a significantly longer history of clinical signs before presentation, whereas clinical signs with CGI were usually more acute in onset. A previous study similarly reported a tendency of LGI to present subacutely or chronically, with a median duration of clinical signs of 3 days.^5^ It is therefore likely that development of a concurrent intestinal lesion triggered an earlier presentation to a referral hospital than would have been the case if the GI was the only lesion. Theoretically, it is also possible that some GI developed secondary to the intestinal lesion (e.g., a displacement obstructing gastric emptying). Considering the large amount of feed material accumulated within the stomach in the face of often very acute intestinal lesions, it seems unlikely that these GI would form spontaneously at the onset of the concurrent lesion.

Gastric impactions can be difficult to diagnose if gastroscopy is not performed and a less invasive method to determine stomach size would be useful. Unfortunately, ultrasonographic assessment of stomach size does not appear to be a sensitive or specific indicator for the presence of GI, and similar findings have been reported previously.^6^ The position of the stomach within the abdominal cavity and its contact with the left abdominal wall might vary, thus making it difficult to appreciate the full size ultrasonographically. Analysis of other ultrasonographic features such as fluid filling have been used to evaluate the equine stomach,^16^ but this feature was difficult to evaluate retrospectively, because of insufficient and variable information recorded by different observers. A prospective investigation regarding the usefulness of ultrasonography in diagnosing GI is necessary, taking additional qualitative assessments into consideration.

Subjective evaluation of gastric impaction size at surgery, but not during gastroscopy, was significantly associated with short‐term survival, and horses with larger impactions at surgery were more likely to be euthanized. It appears that it is easier for surgeons to appreciate the full extent of a GI compared with gastroscopy. During gastroscopy, only the level of filling up to certain landmarks can be appreciated, and this feature may vary with the volume of gas insufflated during each procedure. With gradual feed accumulation, the stomach wall stretches, increasing to double or more of its original size.^17^, ^18^ This dilatation cannot be readily appreciated endoscopically, particularly if the feed extends up to the cardia. A previous study identified marked thickening and fibrosis of the stomach wall, both with and without concurrent intestinal lesions.^17^ In contrast, a descriptive case series described thinning and dilatation of the stomach wall.^18^ Unfortunately, a similar histopathological assessment could not be included in our study. Further comparative assessment of gastric wall changes in GI cases might improve understanding of lesion development.

Contrary to clinical impression, CGI cases took significantly longer to resolve compared with LGI, whereas mean hospitalization time and short‐term survival were similar. Generally, during treatment, the acute concurrent intestinal lesion was addressed first, followed by treatment of the GI. This approach inevitably led to some delay in GI treatment in the CGI group, which could have contributed to the longer treatment times. The CGI cases also experienced recurrence of colic signs more commonly than did LGI cases, presumably because of recurring colic signs related to the concurrent lesion.

In 5 CGI cases, GI developed acutely after surgical correction of intestinal lesions, whereas other CGI cases presented with acute intestinal lesions and GI were detected at surgery. Gastrointestinal inflammation and direct and indirect painful stimuli have been associated with changes in gastrointestinal motility in horses and other species.^19^, ^20^ For example, systemic endotoxin markedly delays gastric emptying in horses,^21^ which may have contributed to GI development postoperatively in our population. Dysmotility caused by decreased neuronal density has been associated with dysautonomia in horses as well as in colonic and cecal obstructive disorders^22^ and could be relevant in recurrent GI. The relationships among systemic disease, the enteric nervous system, and gastrointestinal motility are complex and likely intricately linked, but their importance in the pathogenesis of GI in horses remains to be elucidated.

Against expectation, short and long‐term outcomes were not significantly different between the 2 groups. Gastric rupture was more common in LGI, which could be associated with the longer duration of clinical signs and later diagnosis and treatment of the condition in this group. Although short‐term recurrence of GI was rare, with only 1 horse developing a second GI during hospitalization, 22% of horses experienced at least 1 more GI after discharge, with no differences noted between LGI and CGI cases. This finding is higher than the 11% recurrence previously reported.^5^ With longer follow‐up, particularly for recently hospitalized cases, a higher recurrence rate might be found. Considering that an underlying motility issue is suspected to at least contribute to the development of GI, the high rate of recurrence is not surprising. It also corroborates the assumption that the underlying etiology is similar or the same in both groups. In both groups, many horses required long‐term management changes and only half returned to their previous diet and exercise regimen. However, horses with LGI were significantly more likely to require substantial long‐term dietary changes. The longer duration of the impaction could have led to chronic and possibly irreversible stretching of the stomach wall, or these horses could have suffered from a more severe motility disturbance. However, overall numbers are small, and findings should be confirmed in a larger number of horses to avoid overinterpretation. Feeding a partial‐ or complete‐pelleted diet can be a logistical and financial challenge for horse owners, and can lead to alterations in horses' behavior and gastrointestinal pH.^23^, ^24^ Clients should be well informed of both short‐ and long‐term implications when horses are diagnosed with GI.

Most limitations of our study are associated with its retrospective design. Reporting details differed among clinicians, hospitals, and over time, and information was not always complete. The exact time of diagnostic procedures such as ultrasonography was not always noted in the records. According to hospital protocols, most examinations were carried out within 1 to 2 hours of surgery or euthanasia, but in some cases more time could have elapsed and findings might have been affected. In addition, despite all efforts to rule out other intestinal lesions in the LGI group, some horses might have had unidentified reasons for their colic signs. Treatment details such as the exact volumes of enteral fluids and carbonated soft drinks often could not be obtained from the clinical records. Therefore, comparing the effects of certain treatments was not possible. Our study only included horses located in the south of England and results therefore might not be reflective of a broader international population. However, GI have been reported globally, arguing against distinct regional differences.^5^ The questionnaire required accurate client recall, which may have been difficult, particularly for horses presented early in the 15‐year study period. Several horses were dead, and the reasons for euthanasia were often unclear in the absence of necropsy. Some horses had clinical signs suspicious of GI recurrence but these findings were not confirmed by veterinary examination and gastroscopy.

In conclusion, GI can occur either alone or concurrently with other intestinal lesions and could be underdiagnosed in the presence of other intestinal diseases. Although their clinical presentations and outcomes are similar, gastric rupture and long‐term complications are more likely in LGI. The recurrence of GI might be higher than previously reported.^5^

## CONFLICT OF INTEREST DECLARATION

Authors declare no conflict of interest.

## OFF‐LABEL ANTIMICROBIAL DECLARATION

Authors declare no off‐label use of antimicrobials.

## INSTITUTIONAL ANIMAL CARE AND USE COMMITTEE (IACUC) OR OTHER APPROVAL DECLARATION

Approved by the Social Sciences Research Ethical Review Board (SSRERB) of the Royal Veterinary College. URN: SR2022‐0119.

## HUMAN ETHICS APPROVAL DECLARATION

Authors declare human ethics approval was not needed for this study.
